# *Vital Signs: *Zika-Associated Birth Defects and Neurodevelopmental Abnormalities Possibly Associated with Congenital Zika Virus Infection — U.S. Territories and Freely Associated States, 2018

**DOI:** 10.15585/mmwr.mm6731e1

**Published:** 2018-08-10

**Authors:** Marion E. Rice, Romeo R. Galang, Nicole M. Roth, Sascha R. Ellington, Cynthia A. Moore, Miguel Valencia-Prado, Esther M. Ellis, Aifili John Tufa, Livinson A. Taulung, Julia M. Alfred, Janice Pérez-Padilla, Camille A. Delgado-López, Sherif R. Zaki, Sarah Reagan-Steiner, Julu Bhatnagar, John F. Nahabedian, Megan R. Reynolds, Marshalyn Yeargin-Allsopp, Laura J. Viens, Samantha M. Olson, Abbey M. Jones, Madelyn A. Baez-Santiago, Philip Oppong-Twene, Kelley VanMaldeghem, Elizabeth L. Simon, Jazmyn T. Moore, Kara D. Polen, Braeanna Hillman, Ruta Ropeti, Leishla Nieves-Ferrer, Mariam Marcano-Huertas, Carolee A. Masao, Edlen J. Anzures, Ransen L. Hansen, Stephany I. Pérez-Gonzalez, Carla P. Espinet-Crespo, Mildred Luciano-Román, Carrie K. Shapiro-Mendoza, Suzanne M. Gilboa, Margaret A. Honein

**Affiliations:** ^1^Division of Congenital and Developmental Disorders, National Center on Birth Defects and Developmental Disabilities, CDC; ^2^Oak Ridge Institute for Science and Education, Oak Ridge, Tennessee; ^3^Division of Reproductive Health, National Center for Chronic Disease Prevention and Health Promotion, CDC; ^4^Puerto Rico Department of Health; ^5^U.S. Virgin Islands Department of Health; ^6^American Samoa Department of Health; ^7^Kosrae Department of Health Services; ^8^Republic of Marshall Islands Ministry of Health and Human Services; ^9^Division of Vector-Borne Diseases, National Center for Emerging and Zoonotic Infectious Diseases, CDC; ^10^Division of High-Consequence Pathogens and Pathology, National Center for Emerging and Zoonotic Infectious Diseases, CDC.

## Abstract

**Introduction:**

Zika virus infection during pregnancy causes serious birth defects and might be associated with neurodevelopmental abnormalities in children. Early identification of and intervention for neurodevelopmental problems can improve cognitive, social, and behavioral functioning.

**Methods:**

Pregnancies with laboratory evidence of confirmed or possible Zika virus infection and infants resulting from these pregnancies are included in the U.S. Zika Pregnancy and Infant Registry (USZPIR) and followed through active surveillance methods. This report includes data on children aged ≥1 year born in U.S. territories and freely associated states. Receipt of reported follow-up care was assessed, and data were reviewed to identify Zika-associated birth defects and neurodevelopmental abnormalities possibly associated with congenital Zika virus infection.

**Results:**

Among 1,450 children of mothers with laboratory evidence of confirmed or possible Zika virus infection during pregnancy and with reported follow-up care, 76% had developmental screening or evaluation, 60% had postnatal neuroimaging, 48% had automated auditory brainstem response-based hearing screen or evaluation, and 36% had an ophthalmologic evaluation. Among evaluated children, 6% had at least one Zika-associated birth defect identified, 9% had at least one neurodevelopmental abnormality possibly associated with congenital Zika virus infection identified, and 1% had both.

**Conclusion:**

One in seven evaluated children had a Zika-associated birth defect, a neurodevelopmental abnormality possibly associated with congenital Zika virus infection, or both reported to the USZPIR. Given that most children did not have evidence of all recommended evaluations, additional anomalies might not have been identified. Careful monitoring and evaluation of children born to mothers with evidence of Zika virus infection during pregnancy is essential for ensuring early detection of possible disabilities and early referral to intervention services.

## Introduction

Zika virus infection during pregnancy can cause serious birth defects, including structural abnormalities of the brain and eye (*1*–*7*). As infants with congenital Zika virus infection get older, problems such as epilepsy, vision loss, and developmental delays have been increasingly recognized (*8*). Early identification of and intervention for adverse neurodevelopmental outcomes have been determined to improve cognitive, social, and behavioral functioning and to be cost effective to society in general (*9*–*12*).

The most critical time to intervene and promote optimal brain development is during the first 3 years of life (*9*). To facilitate early identification and intervention, CDC released clinical guidance for the evaluation and management of infants with possible congenital Zika virus infection in January 2016 (*13*). The guidance was based largely on existing guidelines for pediatric health promotion and care (*14*); expert opinion was incorporated from clinicians and researchers with knowledge of congenital infections and of clinical care of infants with birth defects as described in early reports (*15*–*18*). Recommendations for the care and management of infants with possible congenital Zika virus exposure and infants with one or more clinical findings consistent with congenital Zika virus syndrome have remained largely unchanged through subsequent updates (*19*). Standard evaluation* at birth and during each well-child visit is recommended for all infants and young children with possible prenatal Zika virus exposure (*13*,*19*). Laboratory testing for Zika virus is recommended for infants born to mothers with laboratory evidence of confirmed or possible Zika virus infection during pregnancy and for infants with one or more clinical findings consistent with congenital Zika syndrome born to mothers with possible Zika virus exposure, regardless of maternal testing results. In addition to a standard evaluation, infants born to mothers with laboratory evidence of confirmed or possible Zika virus infection during pregnancy should have a cranial ultrasound or other brain imaging and a comprehensive ophthalmologic evaluation performed by age 1 month to detect subclinical brain and eye findings (*19*).

To better understand the effects of Zika virus infection during pregnancy on mothers and children from a national surveillance perspective, CDC collaborated with state, territorial, and local health departments on the U.S. Zika Pregnancy and Infant Registry (USZPIR)^†^ to monitor pregnancy and infant/child outcomes among pregnancies with laboratory evidence of confirmed or possible Zika virus infection (www.cdc.gov/pregnancy/zika/research/registry.html). The USZPIR currently monitors outcomes of approximately 7,300 pregnancies, over 4,800 of which are reported from the U.S. territories and freely associated states^§^ (https://www.cdc.gov/pregnancy/zika/data/pregwomen-uscases.html). This report is the first to provide data on Zika-associated birth defects and neurodevelopmental abnormalities possibly associated with congenital Zika virus infection identified during infancy and early childhood among children aged ≥1 year who were born in the U.S. territories and freely associated states.^§^

## Methods

Pregnancies with laboratory evidence of confirmed or possible Zika virus infection^¶^ and infants resulting from these pregnancies are included in the USZPIR and followed through active surveillance methods (*6*). Data on birth defects and neurodevelopmental outcomes were abstracted from prenatal, birth hospitalization, pediatric, and specialty care medical records using standardized methods and reported to the USZPIR. CDC provided technical assistance to all U.S. territories and freely associated states that reported cases to the USZPIR through the Zika Local Health Department Initiative (https://www.cdc.gov/pregnancy/zika/research/lhdi.html) and the Epidemiology and Laboratory Capacity for Infectious Diseases Cooperative Agreement (https://www.cdc.gov/ncezid/dpei/epidemiology-laboratory-capacity.html). This report includes children who, among pregnancies reported to the USZPIR, 1) were born in U.S. territories or freely associated states; 2) had a date of birth on or before February 1, 2017, and reached age 1 year on or before February 1, 2018; and 3) had follow-up care reported to the USZPIR by June 1, 2018. For the purpose of this analysis, follow-up care was defined as clinical care at age >14 days reported to the USZPIR. Children from multiple gestation pregnancies were counted separately; infants who died during the first year of life were excluded.

Among children who met the definition for reported follow-up care, the percentages who were reported to have received each of the following types of clinical care or evaluations, recommended in CDC clinical guidance, were calculated: 1) neuroimaging (cranial ultrasound, computed tomography, or magnetic resonance imaging) any time after birth; 2) hearing screen by automated auditory brainstem response (ABR) or audiologic evaluation by diagnostic ABR (ABR-based hearing screen or evaluation) any time after birth; 3) ophthalmologic evaluation any time after birth; 4) developmental screening or evaluation at age >14 days; and 5) physical examination, as indicated by reported growth parameters (head circumference, length, or weight) at age >14 days.

Data were reviewed by clinical subject matter experts to identify Zika-associated birth defects and neurodevelopmental abnormalities possibly associated with congenital Zika virus infection (Box). Data for each child were reviewed by at least two reviewers; discrepant review findings were discussed among clinical subject matter experts to reach agreement. Although the category of neural tube defects and other early brain malformations was initially included in the surveillance case definition for Zika-associated birth defects, it was excluded in this report because of growing evidence suggesting a lack of association of these defects with congenital Zika virus infection (*6*,*20*). Postnatal-onset microcephaly detected during follow-up care is distinct from microcephaly detected at birth and is included among neurodevelopmental abnormalities possibly associated with congenital Zika virus infection (Box). Neurodevelopmental findings such as hearing loss, seizures, or swallowing abnormalities consistently documented in reports of infants with possible congenital Zika virus infection were specifically selected for inclusion; however, the broad range of neurodevelopmental abnormalities possibly associated with congenital Zika virus infection necessitates inclusion of less specific but more prevalent categories, such as possible developmental delay. The percentages of these adverse outcomes were calculated among all children born to mothers with laboratory evidence of confirmed or possible Zika virus infection during pregnancy with reported follow-up care, as well as among the subset of children born to mothers with nucleic acid testing (NAT)–confirmed infection during pregnancy, with reported follow-up care.

BOX **Surveillance case classification — children, neonate to 2 years of age, born to mothers with any evidence of Zika virus infection during pregnancy**

**Table:** 

**Zika-associated birth defects:** Selected structural anomalies of the brain or eyes present at birth (congenital) and detected from birth to age 2 years. Microcephaly at birth, with or without low birthweight, was included as a structural anomaly. • **Selected congenital brain anomalies:** intracranial calcifications; cerebral atrophy; abnormal cortical formation (e.g., polymicrogyria, lissencephaly, pachygyria, schizencephaly, gray matter heterotopia); corpus callosum abnormalities; cerebellar abnormalities; porencephaly; hydranencephaly; ventriculomegaly/hydrocephaly. • **Selected congenital eye anomalies:** microphthalmia or anophthalmia; coloboma; cataract; intraocular calcifications; chorioretinal anomalies involving the macula (e.g., chorioretinal atrophy and scarring, macular pallor, and gross pigmentary mottling), excluding retinopathy of prematurity; optic nerve atrophy, pallor, and other optic nerve abnormalities. • **Microcephaly at birth:** birth head circumference ˂3rd percentile for infant sex and gestational age based on INTERGROWTH-21st online percentile calculator (http://intergrowth21.ndog.ox.ac.uk/). **Neurodevelopmental abnormalities possibly associated with congenital Zika virus infection:** Consequences of neurologic dysfunction detected from birth (congenital) to age 2 years. Postnatal-onset microcephaly was included as a neurodevelopmental abnormality. • **Hearing abnormalities**: Hearing loss or deafness documented by testing, most frequently auditory brainstem response (ABR). Includes sensorineural hearing loss, mixed hearing loss, and hearing loss not otherwise specified. Failed newborn hearing screen is not sufficient for diagnosis. • **Congenital contractures:** Multiple contractures (arthrogryposis) and isolated clubfoot documented at birth. Brain anomalies must be documented for isolated clubfoot, but not for arthrogryposis. • **Seizures:** Documented by electroencephalogram or physician report. Includes epilepsy or seizures not otherwise specified; excludes febrile seizures. • **Body tone abnormalities:** Hypertonia or hypotonia documented at any age in conjunction with 1) a failed screen or assessment for gross motor function; 2) suspicion or diagnosis of cerebral palsy from age 1 year to age 2 years; or 3) assessment by a physician or other medical professional, such as a physical therapist. • **Movement abnormalities:** Dyskinesia or dystonia at any age; suspicion or diagnosis of cerebral palsy from age 1 year to age 2 years. • **Swallowing abnormalities:** Documented by instrumented or noninstrumented evaluation, presence of a gastrostomy tube, or physician report. • **Possible developmental delay:** Abnormal result from most recent developmental screening (i.e., failed screen for gross motor domain or failed screen for ≥2 developmental domains at the same time point or age); developmental evaluation; or assessment review by developmental pediatrician. Results from developmental evaluation are considered the gold standard if available. • **Possible visual impairment:** Includes strabismus (esotropia or exotropia), nystagmus, failure to fix and follow at age <1 year; diagnosis of visual impairment at age ≥1 year. • **Postnatal-onset microcephaly:** Two most recent head circumference measurements reported from follow-up care <3rd percentile for child’s sex and age based on World Health Organization child growth standards; downward trajectory of head circumference percentiles with most recent measurement <3rd percentile. Age at measurement was adjusted for gestational age in infants born at <40 weeks’ gestational age through age 24 months chronological age.

A sensitivity analysis to address the concern about possible misclassification of microcephaly at birth** was performed by excluding infants with a birth head circumference measurement indicating microcephaly and no other Zika-associated birth defects, who subsequently had normal neuroimaging and at least two postnatal measurements with a head circumference above the tenth percentile for the infant’s age and sex. Among infants tested after birth for Zika virus with either NAT or serologic tests (immunoglobulin M [IgM]) in serum, urine, or cerebrospinal fluid, the percent positivity is reported.

## Results

**FIGURE 1:**
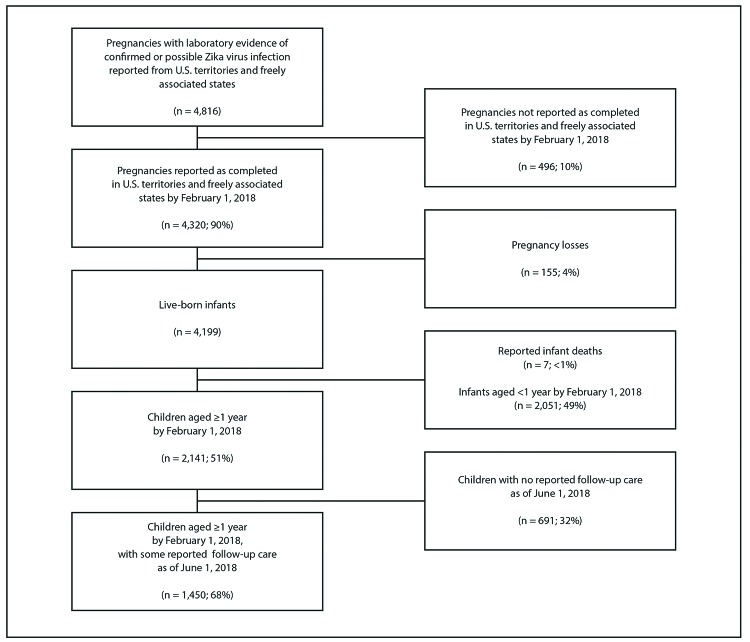
Children born to mothers with laboratory evidence of confirmed or possible Zika virus infection during pregnancy — U.S. Zika Pregnancy and Infant Registry, U.S. territories and freely associated states, February 1, 2017–June 1, 2018*^,†,§,¶,^** * Percentages might not sum to 100 because of rounding. ^†^ Date and location of pregnancy completion were required to document a completed pregnancy in U.S. territories and freely associated states. ^§^ Live-born infants include 4,199 infants from 4,165 pregnancies (includes 34 multiple gestation pregnancies). ^¶^ Of the 691 children with no reported follow-up care as of June 1, 2018, 99 were reported to have moved out of U.S. territories and freely associated states. ** Of the 1,450 children aged ≥1 year by February 1, 2018, with some reported follow-up care by June 1, 2018, 154 were reported to have moved out of U.S. territories and freely associated states.

The U.S. territories and freely associated states reported 4,816 pregnancies with laboratory evidence of confirmed or possible Zika virus infection by June 1, 2018, including 4,320 (90%) completed on or before February 1, 2018; 4,165 (96%) pregnancies resulted in 4,199 live-born infants, and 155 (4%) resulted in a pregnancy loss (Figure 1). Seven infants were excluded who would have reached age 1 year on or before February 1, 2018 and were reported to have died, including three who died during the first 14 days of life. By February 1, 2018, a total of 2,141 (51%) children were aged ≥1 year, 1,450 (68%) of whom had some follow-up care reported to the USZPIR after age 14 days.

**FIGURE 2:**
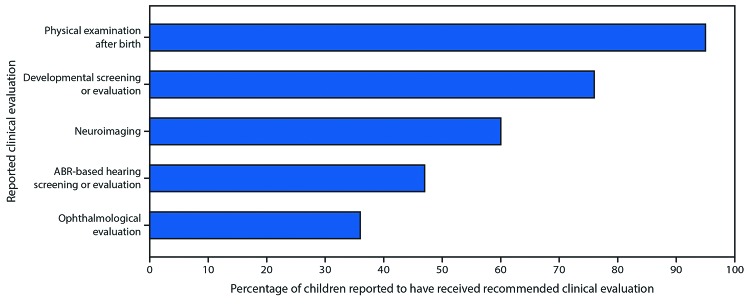
Percentage of children aged ≥1 year born to mothers with laboratory evidence of confirmed or possible Zika virus infection during pregnancy reported to have received recommended clinical evaluations*^,†,§,¶,^** among children with reported follow-up care†† (n = 1,450) — U.S. Zika Pregnancy and Infant Registry (USZPIR), U.S. territories and freely associated states, February 1, 2017–June 1, 2018 **Abbreviation:** ABR = auditory brainstem response. * Physical examination after birth denotes at least one physical examination, indicated by length/height, weight, or head circumference measurements and date of measurements, at age >14 days reported to the USZPIR. **^†^** Developmental screening or evaluation denotes at least one developmental screening or evaluation result at age >14 days reported to the USZPIR. **^§^** Neuroimaging denotes at least one postnatal imaging of the infant head (cranial ultrasound, computed tomography, or magnetic resonance imaging) result reported to the USZPIR. **^¶^** ABR-based hearing screening or evaluation denotes at least one ABR-based hearing screen or evaluation result reported to the USZPIR. Of 1,450 children with reported follow-up care, 96% had at least one hearing screen or evaluation of any kind reported to the USZPIR. ** Ophthalmological evaluation denotes at least one ophthalmological evaluation result reported to the USZPIR. **^††^** Any clinical care at age >14 days reported to the USZPIR.

Among these 1,450 children 1,376 (95%) had at least one physical examination reported after 14 days of life, 1,106 (76%) had at least one developmental screening or evaluation, 864 (60%) had postnatal neuroimaging, and 695 (48%) had at least one ABR-based hearing screen or evaluation. An ophthalmologic evaluation was reported for 522 (36%) children (Figure 2).

Among all 1,450 children with reported follow-up care, 203 (14%) had a Zika-associated birth defect, neurodevelopmental abnormality possibly associated with congenital Zika virus infection identified, or both: 87 (6%) had at least one Zika-associated birth defect, 136 (9%) had at least one neurodevelopmental abnormality possibly associated with congenital Zika virus infection, and 20 (1%) had both (Table). Among the 1,386 (96%) children who did not have microcephaly detected at birth, 822 (59%) received neuroimaging, including 14 (2%) who had at least one brain anomaly identified. In addition, among the 494 (36%) children who received an ophthalmologic evaluation, 12 (2%) had at least one eye anomaly identified. Thus, had these infants not received neuroimaging or ophthalmologic evaluation, 26 brain or eye anomalies in 23 children might have gone undetected.

**TABLE:** Outcomes among children aged ≥1 year from pregnancies with any laboratory evidence of confirmed or possible Zika virus infection (n = 1,450) and with nucleic acid test–confirmed Zika virus infection (n = 943) and with reported follow-up care* — U.S. Zika Pregnancy and Infant Registry (USZPIR), U.S. territories and freely associated states, February 1, 2017–June 1, 2018

Zika-related outcomes	Any laboratory evidence of confirmed or possible Zika virus infection during pregnancy (n = 1,450)^†^ No. (%)	Pregnancies with nucleic acid test–confirmed Zika virus infection (n = 943)^§^ No. (%)
Zika-associated birth defect^¶^	87 (6)	62 (7)
Neurodevelopmental abnormality possibly associated with congenital Zika virus infection**	136 (9)	99 (10)
Zika-associated birth defect and neurodevelopmental abnormality possibly associated with congenital Zika virus infection	20 (1)	17 (2)
**Total with Zika-associated birth defect, neurodevelopmental abnormality possibly associated with congenital Zika virus infection, or both**	**203 (14)**	**144 (15)**
**Microcephaly **		
Microcephaly at birth^††^	64 (4)	44 (5)
Postnatal-onset microcephaly only^§§^	20 (1)	12 (1)
**Total with microcephaly**	**84 (6)**	**56 (6)**

* Any clinical care at age >14 days reported to the USZPIR.

^†^ Includes maternal, placental, or infant laboratory evidence of confirmed or possible Zika virus infection during pregnancy based on presence of Zika virus RNA by a positive nucleic acid test (e.g., reverse transcription–polymerase chain reaction [RT-PCR]), serologic evidence of a Zika virus infection, or serologic evidence of an unspecified flavivirus infection.

^§^ Includes maternal, placental, or infant laboratory evidence of confirmed Zika virus infection during pregnancy based on presence of Zika virus RNA by a positive nucleic acid test (e.g., RT-PCR).

^¶^ Includes Zika-associated birth defect detected from birth to age 2 years with or without neurodevelopmental abnormality possibly associated with congenital Zika virus infection. Zika-associated birth defects include selected congenital brain anomalies (intracranial calcifications; cerebral atrophy; abnormal cortical formation; corpus callosum abnormalities; cerebellar abnormalities; porencephaly; hydranencephaly; ventriculomegaly/hydrocephaly); selected congenital eye anomalies (microphthalmia or anophthalmia; coloboma; cataract; intraocular calcifications; chorioretinal anomalies involving the macula, excluding retinopathy of prematurity; and optic nerve atrophy, pallor, and other optic nerve abnormalities); and/or microcephaly at birth (birth head circumference <3rd percentile for infant sex and gestational age based on INTERGROWTH-21^st^ online percentile calculator (http://intergrowth21.ndog.ox.ac.uk/)).

** Includes neurodevelopmental abnormality possibly associated with congenital Zika virus infection detected from birth to age 2 years, with or without Zika-associated birth defect. Neurodevelopmental abnormalities possibly associated with congenital Zika virus infection include hearing abnormalities; congenital contractures; seizures; body tone abnormalities; movement abnormalities; swallowing abnormalities; possible developmental delay; possible visual impairment; and/or postnatal-onset microcephaly (two most recent head circumference measurements reported from follow-up care <3rd percentile for child’s sex and age based on World Health Organization child growth standards; downward trajectory of head circumference percentiles with most recent <3rd percentile. Age at measurement was adjusted for gestational age in infants born at <40 weeks’ gestational age, through age 24 months chronological age).

^††^ Microcephaly at birth is a subset of Zika-associated birth defects and was defined as birth head circumference <3rd percentile for infant sex and gestational age based on INTERGROWTH-21st online percentile calculator (http://intergrowth21.ndog.ox.ac.uk/)).

^§§^ Postnatal-onset microcephaly is a subset of neurodevelopmental abnormalities possibly associated with congenital Zika virus infection and was defined as two most recent head circumference measurements reported from follow-up care <3rd percentile for child’s sex and age based on World Health Organization child growth standards; downward trajectory of head circumference percentiles with most recent <3rd percentile. Age at measurement was adjusted for gestational age in infants born at <40 weeks’ gestational age, through age 24 months chronological age).

The sensitivity analysis to assess possible misclassification of microcephaly at birth identified 84 (6%) children with microcephaly among the 1,450 children who had follow-up care reported: five infants had microcephaly at birth with brain or eye anomalies identified at birth; 59 had microcephaly at birth with no brain or eye anomalies identified at birth; and 20 infants did not have microcephaly identified at birth but had postnatal identification of microcephaly. The 59 infants with only microcephaly at birth included 15 who had no other Zika-associated birth defects identified during follow-up care, had normal neuroimaging, and had at least two postnatal measurements with a head circumference above the tenth percentile for the infant’s sex and age. Excluding these 15 infants from the 87 with Zika-associated birth defects results in a decrease in the estimated percentage of affected children from 6% to 5%.

Among the 1,450 children whose mothers had laboratory evidence of confirmed or possible Zika virus infection during pregnancy and who had follow-up care reported, 136 (9%) had neurodevelopmental abnormalities possibly associated with congenital Zika virus infection identified (Table). One hundred sixteen (8%) had one or more neurodevelopmental abnormalities possibly associated with congenital Zika virus infection identified, but no Zika-associated birth defects; of these 116 children, 58 (50%) had only possible developmental delay identified, and 25 (22%) had possible developmental delay with at least one other neurodevelopmental abnormality possibly associated with congenital Zika virus infection identified.

Among 943 pregnancies with NAT-confirmed Zika virus infection, 144 (15%) had a Zika-associated birth defect, neurodevelopmental abnormality possibly associated with congenital Zika virus infection identified, or both: 62 (7%) had at least one Zika-associated birth defect identified. Ninety-nine (10%) had at least one neurodevelopmental abnormality possibly associated with congenital Zika virus infection identified, and 17 (2%) had both. 

Among the 1,450 children in this analysis, 607 (42%) did not receive testing for Zika virus infection in serum, urine, or cerebrospinal fluid. Among the 843 (58%) who did receive testing, 32 (4%) tested positive by either NAT or IgM (four of 69 tested by NAT only; zero of 18 tested by IgM only; and 28 of 756 tested by both NAT and IgM tested positive by either NAT or IgM). Zika-associated birth defects or neurodevelopmental abnormalities possibly associated with congenital Zika virus infection were identified in children with positive Zika virus IgM or NAT, negative IgM and NAT, and in those who did not receive testing.

## Conclusion and Comments

A total of  1,450 children aged ≥1 year were born to mothers with laboratory evidence of confirmed or possible Zika virus infection during pregnancy in the U.S. territories and freely associated states and were reported to the USZPIR. Among these children, approximately one in seven (14%) were identified during infancy or early childhood as having either a Zika-associated birth defect, a neurodevelopmental abnormality possibly associated with congenital Zika virus infection, or both.

The 6% with Zika-associated birth defects in this report can be viewed in the context of the previously published baseline frequency of brain and eye abnormalities potentially related to Zika virus infection. Before the introduction of Zika in the Region of the Americas the baseline frequency of brain and eye abnormalities potentially related to Zika virus infection among live-born infants was approximately 0.16% (*21*), suggesting a more than 30-fold increase over baseline. 

Among all children aged ≥1 year by February 1, 2018, 68% had some follow-up care reported to the USZPIR. Of these children, 95% had at least a physical examination, 76% had developmental screening or evaluation, and 60% had neuroimaging. Approximately one half of the children (48%) had an ABR-based hearing screen or evaluation, and approximately one third of the children (36%) had an ophthalmologic evaluation reported to the USZPIR. Because the full spectrum of adverse outcomes related to congenital Zika virus infection is not yet known, careful monitoring and evaluation of children born to mothers with laboratory evidence of confirmed or possible Zika virus infection during pregnancy is essential for ensuring early detection of possible disabilities and early referral to intervention services that might improve outcomes. For example, with early identification of vision problems, a prescription for corrective eyeglasses might be beneficial to a child’s development (*12*). Among children without microcephaly detected at birth, brain or eye anomalies might have gone undetected without neuroimaging or ophthalmologic evaluation.

Many infants did not have Zika virus testing results reported. This could be because of changing recommendations for laboratory testing of infants born to mothers with laboratory evidence of confirmed or possible Zika virus infection during pregnancy (*19*). Among infants with testing reported, only 4% tested positive for Zika virus infection by IgM or NAT. In addition, limitations of laboratory testing for Zika virus have been previously described (*19*); Zika virus RNA is only transiently present in body fluids; thus, a negative NAT result does not rule out infection. Zika virus-associated birth defects and neurodevelopmental abnormalities possibly associated with congenital Zika virus infection also were identified in children with negative Zika virus NAT or IgM test results. These finding are consistent with other reports of infants with clinical findings suggestive of possible congenital Zika syndrome but with negative laboratory results (*2*,*20*,*22*).

Microcephaly is challenging to monitor accurately as an outcome because it is difficult to reliably measure head circumference in a newborn, it can be affected by inaccuracies in estimated gestational age, and it does not distinguish between a small head size related to underlying pathology and one that will subsequently exhibit typical brain development (*3*). The sensitivity analysis suggests that the number of infants with Zika-associated birth defects could be a modest overestimate.

This is the first analysis assessing neurodevelopmental abnormalities possibly associated with congenital Zika virus infection in addition to Zika-associated birth defects among children born to mothers in the U.S. territories and freely associated states with laboratory evidence of confirmed or possible Zika virus infection during pregnancy. Although there are large cohort studies monitoring pregnancies with and without Zika virus infection in several countries, the data in this report come from the largest cohort of children born to mothers with laboratory evidence of confirmed or possible Zika virus infection during pregnancy in the world who are currently being monitored as part of an enhanced surveillance system.

Whereas the cohort size is a strength of this analysis, the findings in this report are subject to at least five limitations. First, the data are limited to evaluations and clinical care received and reported to the USZPIR. The recommended services might not have been available to all children, and among those with reported follow-up care, information was limited for some children. In addition, data are limited to clinical records reported to the USZPIR; collecting these data are challenging because children might be seen in various outpatient settings and by multiple providers. To alleviate this barrier, territorial and state jurisdictions made extensive efforts to actively follow up, abstract, and report available data; CDC also provided substantial technical assistance. Second, it is possible that children with recognized health problems might have received follow-up care more frequently than did those without identified health problems, which might lead to an overestimate of the percentage of children with Zika-related health problems. Third, estimates of the baseline frequencies of neurodevelopmental abnormalities among very young children are available only for a few of the specific abnormalities; the lack of an appropriate comparison group limits assessment of whether the prevalence of reported neurodevelopmental abnormalities in the U.S. territories and freely associated states among children born to mothers with laboratory evidence of confirmed or possible Zika virus infection during pregnancy is an increase over baseline levels. Fourth, given the potential persistence, cross-reactivity, or nonspecific reactivity of IgM, some mothers included in the USZPIR might not have been infected with Zika virus during their pregnancy. For this reason, an analysis of child outcomes restricted to pregnancies with NAT-confirmed Zika virus infection was included, and similar percentages of children with a Zika-associated birth defect, a neurodevelopmental abnormality possibly associated with congenital Zika virus infection, or both were found. Finally, it might be difficult to distinguish between birth defects and neurodevelopmental abnormalities that might be causally linked to congenital Zika virus infection and those that might be attributable to unrelated causes; thus, this report describes occurrences without attributing causation.

Despite the limitations, this report extends understanding about the impact of congenital Zika virus infection. Whereas approximately 6% of children with congenital Zika virus exposure have Zika-associated birth defects, more children have neurodevelopmental abnormalities possibly associated with congenital Zika virus infection, identified during follow-up care, albeit without an appropriate comparison group on the baseline prevalence of these neurodevelopmental abnormalities among very young children. Given that most children did not have evidence of all recommended evaluations according to data reported to the USZPIR, additional unidentified anomalies might exist in this population. Furthermore, it is recognized that there were substantial disruptions to the provision of clinical care in Puerto Rico and the U.S. Virgin Islands related to hurricanes in 2017 (*23*); many families also were internally displaced or left the affected territories, potentially resulting in fewer follow-up care data reported to the USZPIR. Children who were most affected by Zika virus infection during pregnancy might have been either more or less likely to be displaced after hurricanes; there is no specific information on the impact of this displacement in these estimates. However, jurisdictional staff members attempted to find families and link them to the USZPIR in their new jurisdiction.

It is essential that health care providers who care for children have access to information regarding maternal exposure to Zika virus infection during pregnancy. This will improve the identification of children born to mothers with laboratory evidence of confirmed or possible Zika virus infection during pregnancy so that they can receive recommended postnatal evaluations. Zika virus transmission is far less prevalent in the Americas in 2018 than during 2015–2017 (https://www.cdc.gov/zika/reporting/case-counts.html); however, information about this cohort of children can inform and guide future responses to outbreaks of Zika virus that will inevitably occur among susceptible populations and disproportionately affect pregnant women and their children.

**Summary**

**What is already known about this topic?**

Zika virus infection during pregnancy can cause serious birth defects and might be associated with neurodevelopmental abnormalities.

**What is added by this report?**

Among children aged ≥1 year born in U.S. territories and freely associated states to mothers with laboratory evidence of confirmed or possible Zika virus infection during pregnancy and who had follow-up care reported, 6% had a Zika-associated birth defect, 9% had ≥1 neurodevelopmental abnormality possibly associated with congenital Zika virus infection, and 1% had both.

**What are the implications for public health practice?**

Given the potential benefits from interventions during early critical periods of infant development, health care providers should share information on maternal Zika virus exposure and closely monitor child health and development.

## Footnotes

* Standard evaluation includes a comprehensive physical exam, including growth parameters; newborn hearing screen, preferably with automated auditory brainstem response (ABR); developmental monitoring and screening using validated screening tools recommended by the American Academy of Pediatrics (https://www.aap.org/en-us/advocacy-and-policy/aap-health-initiatives/Screening/Pages/Screening-Tools.aspx); and vision screening as recommended by the American Academy of Pediatrics Policy Statement “Visual System Assessment in Infants, Children, and Young Adults by Pediatricians” (http://pediatrics.aappublications.org/content/137/1/e20153596). Infants should be referred for automated ABR by age 1 month if the newborn hearing screen was passed using only otoacoustic emissions methodology.

† The U.S. Zika Pregnancy and Infant Registry (USZPIR) refers to the Zika Pregnancy and Infant Registries implemented in all U.S. states, the District of Columbia, all U.S. territories, and U.S. freely associated states. The USZPIR is an enhanced surveillance system that collects data on pregnancy and infant/child outcomes in pregnancies with laboratory evidence of confirmed or possible Zika virus infection. In Puerto Rico, the USZPIR is also known as the Zika Active Pregnancy Surveillance System (ZAPSS). Children are followed through age 36 months in Puerto Rico and through age 24 months in other U.S. territories, freely associated states, and U.S. states.

§ U.S. territories and freely associated states reporting cases included American Samoa, Federated States of Micronesia, Marshall Islands, Puerto Rico, and the U.S. Virgin Islands.

¶ Maternal laboratory evidence of confirmed or possible Zika virus infection was defined as 1) Zika virus infection detected by a Zika virus RNA nucleic acid test (NAT) (e.g., reverse transcription–polymerase chain reaction [RT-PCR]) on any maternal, placental, fetal, or infant specimen (referred to as NAT-confirmed) or 2) detection of recent Zika virus infection or recent unspecified flavivirus infection by serologic tests on a maternal, fetal, or infant specimen (i.e., either positive or equivocal Zika virus immunoglobulin M [IgM] and Zika virus plaque reduction neutralization test [PRNT] titer ≥10, regardless of dengue virus PRNT value; or negative Zika virus IgM, and positive or equivocal dengue virus IgM, and Zika virus PRNT titer ≥10, regardless of dengue virus PRNT titer). Infants with positive or equivocal Zika virus IgM are included, provided a confirmatory PRNT has been performed on a maternal or infant specimen. The use of PRNT for confirmation of Zika virus infection, including in pregnant women and infants, is not routinely recommended in Puerto Rico; dengue virus is endemic and cross-reactivity is likely to occur in most cases (https://www.cdc.gov/zika/laboratories/lab-guidance.html). In Puerto Rico, detection of a positive Zika IgM result in a pregnant woman, fetus, or infant (within 48 hours after delivery) was considered sufficient to indicate possible Zika virus infection.

** Microcephaly was defined as head circumference at delivery <3rd percentile for infant sex and gestational age, regardless of birth weight. When multiple head circumference measurements were available, the majority of those measurements had to be <3rd percentile for a designation of microcephaly. A clinical diagnosis of microcephaly or mention of microcephaly or small head in the medical record was not required. (https://www.cdc.gov/pregnancy/zika/data/pregnancy-outcomes.html).
